# Leveraging telemedicine to improve MNCH uptake in Kenya: a community-based hybrid model

**DOI:** 10.3389/fdgth.2025.1668776

**Published:** 2026-01-19

**Authors:** Edna Anab, Tabither Gitau, Erick Yegon, Nzomo Mwita, Marlyn Ochieng, Alice Koimur, Rhonnie Omondi, Stephen Smith, Harriet Andrews, David Oluoch, Rosebella Amihanda, Moses Lwanda, Erina Makhulo, Godfrey Sakwa, Phanice Akinyi

**Affiliations:** 1 Innovations Department, Living Goods, Nairobi, Kenya; 2 Performance Evidence & Insights, Living Goods, Nairobi, Kenya; 3 Digital Health & Innovation, Health X Africa, Nairobi, Kenya; 4 Partnerships Advocacy and Communication, Living Goods, Nairobi, Kenya; 5 Programs, Living Goods, Nairobi, Kenya

**Keywords:** telemedicine, maternal health, newborn health, child health, community health workers, digital health, healthcare access, service utilization

## Abstract

**Background:**

Kenya faces significant challenges in providing adequate access to maternal, newborn, and child health services, particularly in remote and underserved areas. Limited infrastructure, healthcare worker shortages, and financial constraints hinder access to timely, essential care. As health systems continue to face increasing demands, Telehealth solutions offer a promising approach to bridging geographical gaps and improving access to timely and essential healthcare services. By leveraging technology, telehealth can connect patients in remote areas with healthcare providers, enabling virtual consultations, remote monitoring, and timely interventions.

**Aim:**

This study evaluated the “Better Data for Better Decisions: Telehealth” initiative, funded by The Children's Investment Fund Foundation (CIFF) and implemented by Living Goods and in partnership with Health X Africa. The innovation aimed to integrate telehealth into the Community Health Promoter framework to improve MNCH outcomes, focusing on antenatal and postnatal care. The specific objectives included increasing uptake of antenatal and postnatal care, improving the efficiency of primary healthcare delivery, and influencing relevant policies.

**Setting:**

The study was conducted in Teso North, Busia County, Kenya, targeting ten community health units.

**Method:**

A mixed-methods quasi-experimental design was employed, incorporating key informant interviews, focus group discussions, and routine health record reviews. Data collection involved desk reviews, field data collection, and virtual data collection across three phases. Quantitative data were analyzed in Stata® 15 and R 4.5.1 using descriptive, inferential, and GEE models, while qualitative data were coded and analyzed in Dedoose using a constant comparative method.

**Result:**

The project exceeded its registration targets, enrolling 388 households and 551 clients. Of the registered clients, 50% engaged in consultations with Health X doctors via the hotline, which emerged as the most preferred service channel, used by approximately 88% of Telehealth platform users. The intervention positively impacted the frequency of postnatal care (PNC) touchpoints and identified at-risk women based on nutritional indicators. The average number of PNC visits within six weeks postpartum was significantly higher in the intervention sites (mean: 4.99 visits) compared to control units (mean: 3.96 visits; *p* = 0.003). The big wins for impact were identifying and escalating care, including completion of referrals for dangers signed in newborns, supporting positive behaviour change and improving access to clinical care in the last mile.

**Conclusion:**

Integrating telemedicine into the CHW framework shows promise for improving access to and engagement with postnatal care services in underserved areas of Kenya. The hybrid model, combining virtual consultations with community-based CHW support, effectively leveraged technology and existing health infrastructure. Further research is needed to assess the impact on healthcare efficiency and policy influence fully. These findings present a compelling case for policymakers to scale telehealth as a core element of Kenya's MNCH strategy. Part of the work led to supporting the MOH in developing Telemedicine Policy and Guidelines for Kenya.

## Introduction

Kenya, like many low- and middle-income countries, faces significant challenges in achieving optimal maternal, newborn, and child health outcomes. A key barrier is limited access to quality healthcare services, particularly in remote and underserved areas. This is further compounded by factors such as inadequate infrastructure, shortages of healthcare professionals, and financial constraints. Traditional healthcare delivery models often struggle to effectively reach these marginalized populations, resulting in disparities in health outcomes. Telehealth has emerged as a transformative tool in healthcare, offering innovative solutions to bridge access gaps, particularly in low-resource settings (1). The COVID-19 pandemic accelerated the adoption of digital health solutions, including telemedicine, as physical distancing measures disrupted traditional service delivery (2). Telemedicine has proven highly effective in managing chronic conditions, especially diabetes. It has been linked to better patient outcomes and reduced healthcare costs (3). According to the 2022 Kenya Demographic and Health Survey (KDHS) (4), the neonatal mortality rate (NMR) has declined to 21 deaths per 1,000 live births, down from 31 in 2014. Similarly, the infant mortality rate (IMR) decreased to 32 deaths per 1,000 live births from 39 in 2014. Maternal and neonatal health outcomes in Kenya remain a public health priority, with the country facing high maternal mortality rates and only modest declines in neonatal deaths. These challenges are particularly acute in underserved regions, where traditional care models struggle to reach vulnerable populations. The first 1,000 days of life represent a critical window for intervention, yet many mothers and newborns continue to lack timely, quality care, especially during the postnatal period.

Globally, evidence increasingly positions telehealth within the broader framework of digital transformation in healthcare, emphasizing not only its operational benefits but also its role in reshaping user engagement, workforce capability, and innovation ecosystems (5). Studies on digital adoption in Indonesia and South Africa demonstrate that leadership commitment, digital literacy, and patient-centric design are key determinants of successful telehealth integration. Similarly, research on consumer sentiment in telemedicine underscores that digital adoption is influenced as much by social trust and user experience as by technology itself (6). Telemedicine refers specifically to the provision of clinical services at a distance, enabling healthcare professionals to assess, diagnose, and manage patients through digital communication channels (2). Telehealth provides an opportunity to expand essential services to communities that have limited access to conventional healthcare, especially those living in hard-to-reach areas. Historically, remote care began with basic telegraph and telephone exchanges, but it has expanded considerably with the rise of video-based consultations, sensor-enabled devices, and AI-driven tools. These advancements have shifted remote care from isolated virtual visits to comprehensive digital ecosystems that support multiple aspects of healthcare delivery (7). These insights highlight the importance of community-level mediation and continuous engagement in digital health programs elements that are often underexplored in low-resource contexts.

In Kenya, Living Goods (LG), funded by Children's Investment Fund Foundation (CIFF) project, sought to operationalize this concept through a hybrid telehealth model. The approach combined virtual consultations with physical follow-up and triage by trained Community Health Workers (CHWs), creating a bridge between households and clinical care. The pilot intervention, conducted in Malaba, Busia County, aimed to assess the feasibility, effectiveness, and sustainability of integrating telemedicine into MNCH service delivery. This research is significant for two reasons. First, it addresses an evidence gap in understanding how hybrid telehealth models can enhance maternal and newborn care in fragile health systems. Second, it situates telemedicine within Kenya's ongoing digital transformation agenda, aligning with national strategies for community health digitization and the Sustainable Development Goals (SDGs) on reducing preventable maternal and neonatal deaths. By examining behavioral, contextual, and operational determinants of telehealth use, the study contributes to global discourse on how digital innovations can advance equitable, patient-centered, and scalable health systems.

## Methodology

### Study design

**Figure 1:**
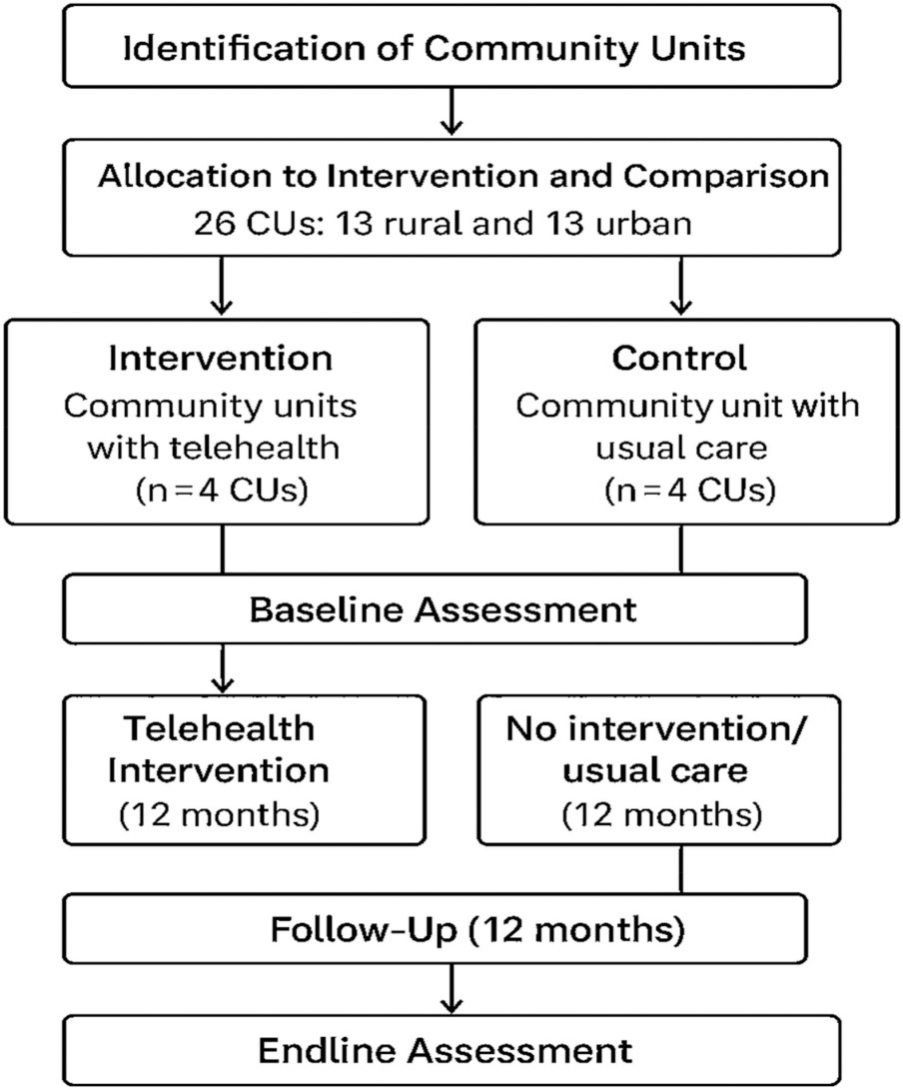
Description of the study design (quasi-experimental design).

This study employed a quasi-experimental design with a mixed methods approach to evaluate the impact of the intervention. Figure 1 visually represents the study design. It illustrates the selection of CUs from the larger population and their assignment to intervention and comparison groups. This was a non randomized implementation evaluation where CUs were purposively selected in consultation with the Department of Health in Busia County to ensure representativeness and feasibility. The matching process is based on size, population characteristics, workforce availability, and health facility spread to minimize pre-existing differences that could confound the results. Table 1 outlines the digital communication channels and measurement tools used in the study. These included SMS, a hotline, USSD, and IVR platforms. The study's aim was to generate practical evidence on how well each channel performs in harder-to-reach, remote settings, and to identify which tools are most appropriate for different operational needs.

**Table 1:** Digital tools and outcome mapping.

Tool/Channel	Functionality	Outcome area supported
IVR (Interactive Voice Response)	Danger sign self-screening for mothers and infants	Early identification and escalation of high-risk cases
USSD	Client-initiated registration and basic interaction	Empowering user-initiated care-seeking behavior
SMS (One-way & Two-way)	Health education reminders, referral notifications, PNC/ANC alerts	Improved attendance and adherence to care schedules
Hotline (Toll-Free Calls)	Real-time consultation with doctors for maternal and infant issues	Increased access to timely clinical guidance
CHW physical visits	Household engagement, follow-up, and support	Continuity of care, community mobilization

### Ethical approval and consent

During project implementation, all participants provided informed consent, either verbally or in writing, depending on literacy levels. CHWs were trained on ethical practices, including data confidentiality and consent procedures. For minors aged 15–17 involved in the intervention (as eligibility was later expanded), assent was obtained alongside guardian consent in accordance with ethical guidelines. Additionally, study ethical approval was sought from the JKUAT ethical review committee.

### Study setting

The study was conducted in Teso North Sub County, Busia County, Kenya. Teso North was chosen due to the existing healthcare access challenges, especially concerning antenatal and postnatal care. The evaluation focused on 10 Community Health Units within Teso North. Six of these CHUs (3 peri-urban and 3 urban) were assigned to the intervention group, while 4 served as the comparison group.

As shown in Figure 1, ten community units were selected from a total of 26 in Teso North, Busia County, Kenya. Six of these CUs were assigned to the intervention group, while the remaining four served as the comparison group.

### Study population and sampling

The study population consisted of households within the purposively selected Community Units (CUs) in Teso North, Busia County, Kenya, that had children under 6 weeks old and/or pregnant women of reproductive age (18–49 years). All eligible households within the chosen CUs were included in the study, forming the total sampling frame.

A cluster sampling approach was used, where the CUs represented the clusters, and all eligible households within those clusters were included. This targeted sampling ensured the evaluation focused on the population most relevant to postnatal care interventions. The study included 129 surveys completed by mothers (20 telehealth service non-users and 109 users of the intervention) and seven FGDs with mothers. Additional FGDs were conducted with CHWs and local government staff to gain broader perspectives on the intervention's impact.

### Intervention description

The project aimed to design and implement an integrated virtual and physical 24-hour triage solution, allowing clients to self-screen and be directed towards appropriate care pathways. The goal was to enhance care-seeking behaviours, reduce delays in care, and improve efficiency in delivering care.

This intervention integrated a telemedicine solution into an existing community health platform, with the goals of:

Improving the uptake of essential healthcare services, specifically postnatal care.
Increasing the efficiency of primary healthcare delivery by reducing the need for in-person visits.
Ultimately, influencing national and county-level policies to create a supportive environment for telemedicine innovations.

The intervention incorporated Interactive Voice Response ^1^(IVR) for self-screening of danger signs for mothers and infants, contributing to early identification of danger signs and escalation for better health outcomes. Additional SMS for reminders and notification of referrals from Health X to the CHWs and supervisors. It operated alongside the routine service delivery model of CHWs, offering an additional layer of support and access to care. This hybrid approach leveraged both technology and existing community health infrastructure to address challenges in accessing postnatal care services (Refer to Figure 3).

### Study phases

The project followed a 24-month schedule organized into three phases: a six-month period of exploration and co-design, six months of prototyping and early testing, and a twelve-month pilot implementation and evaluation phase. The evaluation itself was carried out in three stages, as outlined in Table 2: *Telehealth Intervention Timeline and Rollout Phases*.

**Table 2:** Telehealth intervention timeline and rollout phases.

Phase	Timeline	Key activities	CHUs involved	Tools/Channels deployed
Phase 1: Design & Co-Design	February–July 2023	Formative research, feasibility assessments, partner selection (HealthX Africa), design approvals	N/A	Planning only
Phase 2: Pilot Rollout – Phase I	June–October 2023	Launch with 17 CHWs in two CHUs (Moding and Komiriai); IVR, hotline, SMS rollout	Moding, Komiriai	USSD, Hotline, One-way SMS, IVR
Phase 2: Pilot Rollout – Phase II	November 2023–February 2024	Expansion to 22 CHWs in two more CHUs; wider IVR rollout, expanded eligibility (15+ years)	Ikapolok, Onyunyur	Expanded IVR, Hotline, Personalized SMS
Phase 3: Optimization & Scale	March–September 2024	Two-way SMS added, standardized SMS schedule; evaluation of engagement, referrals, and usage	All 4 pilot CHUs	Two-way SMS, Standardized Messaging
Endline Evaluation	September 2024	Final data collection, analysis of uptake, impact, and referral completion	All Intervention Sites	Monitoring tools, clinical data, FGDs & KIIs

#### Phase one

##### Design phase

This phase entailed formative research, sector landscaping, and early-stage exploratory research on compatibility and feasibility of modalities. Additionally, this phase entailed scoping and selection of a partner in technology and telemedicine service provision, settling on HealthX Africa.

#### Phase two

##### Innovation implementation

This phase entailed submission of project design documents to the donor (CIFF) for sign off and seeking ethical approvals. This was followed by the launch of Phase I of the intervention, where 17 CHWs in the first two Community Units (Moding and Komiriai in June 2023, with review and design pivots. The pilot went live with the USSD (English & Swahili), hotline, and SMS channels. Phase II, spanning from November 2023 to February 2024, engaged an additional 22 CHWs from Ikapolok and Onyunyur Community Health Units. In terms of the intervention, this phase included the full deployment of IVR, extending eligibility to include clients aged 15 years and above. Phase III began in March to September 2024 with design pivots where the Two-way SMS was launched in addition to personalized SMS and a standardised schedule of health information SMS.

#### Phase three

#### Endline evaluation

The endline evaluation was conducted in September 2024.

**Figure 2:**
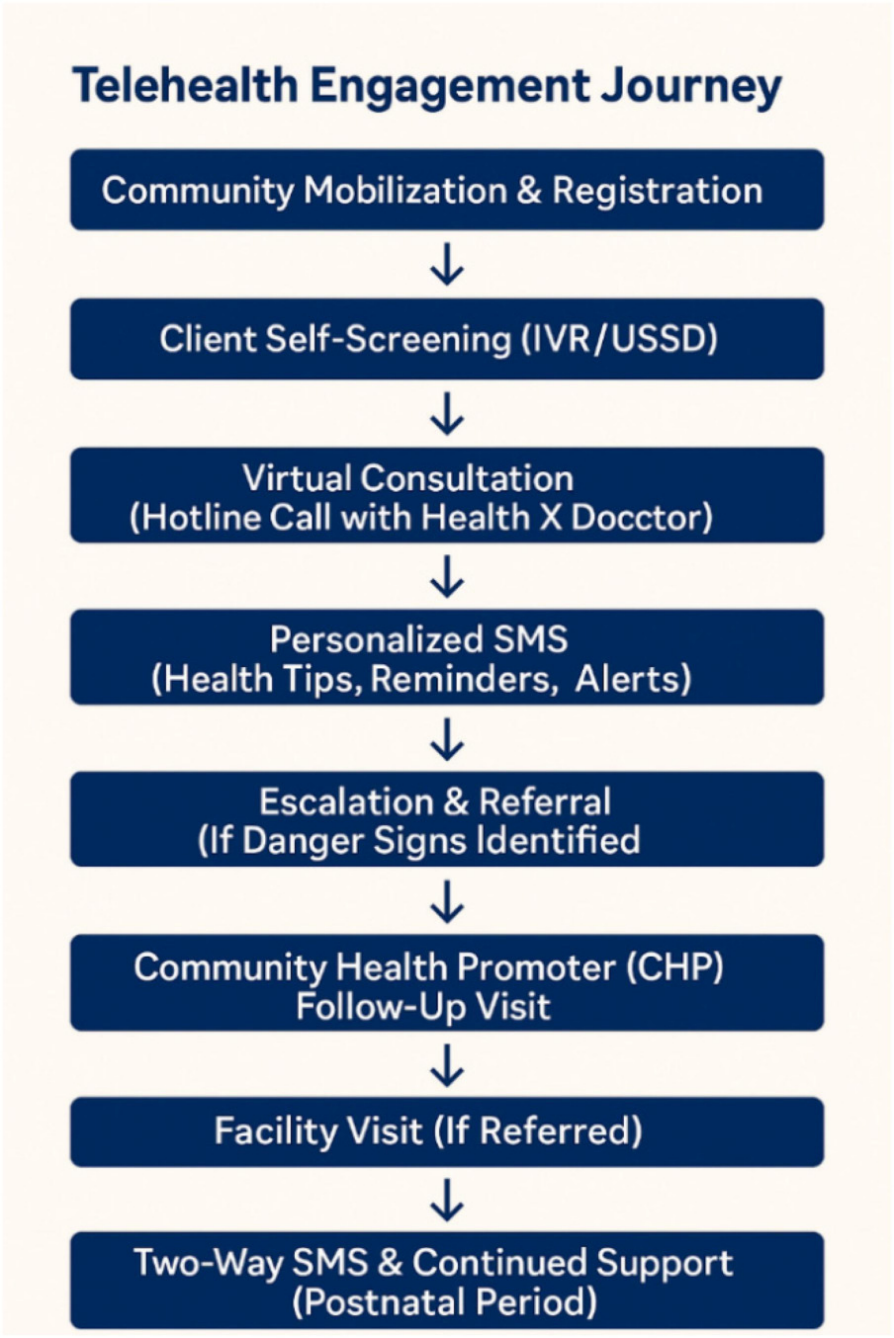
Telehealth engagement journey.

The *Telehealth Engagement Journey* (Figure 2) outlines the sequential process through which clients interact with the hybrid telehealth model. This framework was not merely illustrative but empirically derived from pilot implementation data, user feedback, and CHP field experience. The journey reflects how clients transition from initial mobilization and digital self-screening through virtual consultations, automated SMS follow-ups, and in-person support where required. By combining digital and community touchpoints, the process ensures timely escalation, referral, and continued postnatal engagement, illustrating a pragmatic pathway for integrated telehealth delivery at the community level.

### Data collection

The data collection for this study employed a mixed-methods approach, gathering both quantitative and qualitative data across three distinct phases:

#### Desk review

This phase was conducted between July 15th and September 6th, 2020, and involved reviewing project documents, Health X databases, and Local Government databases. The purpose of this review was to establish background and context for the study, as well as to triangulate findings from the primary data collection activities. The desk review culminated in an inception report outlining the finalized evaluation methodology, timelines, and data collection tools.

#### Field & virtual data collection

This phase took place from September 9th to 23rd, 2024, utilizing a stratified, criteria-based, purposive sampling method to select participants. Several data collection methods were employed:

#### Key informant interviews

Semi-structured interviews were conducted with various stakeholders, including county and sub-county health officers, mothers participating in the intervention, a local government telehealth champion, 4 County and Sub County Ministry of Health staff, nurses from referral facilities, and Health X staff. Tailored interview guides were developed for each stakeholder group to ensure relevant and focused data collection.

#### Focus group discussions

A total of 11 FGDs were held, involving 168 primary beneficiaries (ANC and PNC mothers), and 38 Community Health Workers. Participants were segmented by telehealth service user engagement levels (high, medium, and low) for both mothers and CHWs.

#### Monitoring program data

Project performance indicators related to service utilization and health outcomes were analyzed using data spanning from July 15th to September 30th, 2024. This quantitative data was extracted from project data.

#### Health X consultation records review

This data was use to assess uptake levels and patterns across users over the intervention period to facilitate consistent learnings of the gaps across platforms and course correction to improve access.

### Data analysis

Both quantitative and qualitative data analysis techniques were applied. Quantitative data were analyzed using Stata® version 15 and R version version 4.5.1 (2025-06-13 ucrt), employing descriptive, exploratory, and inferential statistics. Impact on on-time PNC visits were quantified using the difference in difference, generalized Cohen's d effect size. To examine determinants of receiving on time postnatal care, the averaged logistic regression model using generalized estimating equations (GEE) with an AR(1) correlation structure was fitte. For qualitative data, the audio recordings were transcribed and imported into Dedoose for coding and further analysis. Analysis across all transcripts was conducted using a constant comparative method to identify emerging themes and their repetitions and variations.

## Results

This section summarizes the key findings on the hybrid virtual telehealth and Community Health Worker intervention in Teso North, Kenya, focusing on user registration, adoption, CHW Influence in the intervention uptake, service uptake, impact, nutrition, and sustainability.

### User registration and client profile

The hybrid model and virtual platform supported 388 households, reaching 551 clients (388 mothers and 163 infants) through 39 Community Health Providers. Program data indicated that the majority of the registered mothers were young adults aged between 20 and 29 years at 59% (*n* = 228), with the majority enrolling during their second trimester at 27% (106 women). 113/129 (88%) of registered women surveyed were married, while 12% (16) self-reported as single mothers. Regarding education, more than half of the mothers had attained primary and secondary education at 62(48%) and 50(39%), respectively. By the end-line study in September 2024, 77% (99/129) had delivered. Among those surveyed, 88% (113/129) were married and 12% (16) were single mothers, with most having primary, 48% (62), or secondary, 39% (50) education. It was critical to note that almost half of these mothers had no source of income at 48% (*n* = 62), translating to 50% of the mothers reporting to have no independent income whatsoever (*n* = 65). By the end-line study in September 2024, a significant majority (77%, 99 women) had given birth, while 60 eligible women (either pregnant or within six weeks postpartum) remained enrolled at the time of reporting. These results highlight telehealth's potential to engage women earlier in their pregnancy, despite the project's primary focus being on postnatal care.

### Adopters: client profile and frequency of use

Almost half of the registered women and the same proportion of infants utilized the Telehealth services. High adoption of Telehealth services was noted in Moding and Komiriai CUs, which were the Phase one intervention sites. While the two phase two sites, almost at par with about 15% contribution each to the total adoption rates. High adoption was noted in Moding (Phase 1) and Komiriai (Phase 2) CUs, both accounting for 58% (111/189) of all adopters (Table 3).

**Table 3:** Registered clients and adoption rate by community unit.

Telehealth adoption rate by community unit				
Community unit	CU description	Women	Infants	Duration of implementation
Moding	Rural	52/136 (38%)	34/61 (56%)	Phase 1: June '23
Komiriai	Urban	59/114 (52%)	27/53 (51%)	Phase 1: June '23
Ikapolok	Peri-urban	44/73 (60%)	7/30 (23%)	Phase 2: Nov '23
Onyunyur	Rural	34/61 (56%)	11/18 (61%)	Phase 2: Nov '23
Non-users		178	76	
Missing		21	8	
**Total**		**388**	**163**	

Bold values indicate a total of 388 mothers and 163 infants were enrolled in the study.

According to Health X data 49% of women took up the service (engaged with the clinical hotline) at least once after registration. 85% (109/129) of women surveyed reported to be users of telehealth services (Table 4). Between June 2023 and August/September adoptions rates (proactive engagement by the register user at least once after registration was 49% (189/388 women registered). The definition of adoption (measured by clinical calls with HealthX) is as follows:

High adopters: Engagement 4 times or more after registration were classified as high users
Medium adopters: engagement 2–3 times after registration
Low Adopters: Engagement once after registration
No adopters: zero engagement after registration.

**Table 4:** Frequency of use by community unity.

Frequency of use by community unity (June 2023–September 2024)						
Community unit	High	Medium	Low	Non-users	Total	Total users
Moding	11 (3%)	19 (5%)	22 (6%)	69 (18%)	121 (33%)	52 (13%)
Komiriai	3 (1%)	26 (7%)	30 (8%)	51 (13%)	110 (30%)	59 (15%)
Ikapolok	8 (2%)	22 (6%)	14 (4%)	33 (9%)	77 (21%)	44 (11%)
Onyunyur	6 (2%)	12 (3%)	16 (4%)	25 (6%)	59 (16%)	34 (9%)
Missing data					21 (5%)	
	28 (8%)	79 (22%)	82 (22%)	178 (49%)	100%	189 (49%)

CHWs observed that high adopters of the virtual health service were typically individuals with a high perceived need, such as first-time mothers, adolescents, or those with a history of obstetric complications. They also tended to have slightly higher literacy and education levels, owned phones, or had supportive partners willing to share their phones and access to the service. A key driver of adoption was the quality of virtual customer service. Many users reported receiving more personalized care, longer consultations, and access to broader medical expertise than they typically received from local health facilities or CHWs.

In contrast, non-adopters often cited limited time and low perceived value of the service, believing they would be referred to a facility for care regardless. CHWs reported that these mothers often had low literacy levels, poor initial customer service experiences (such as missed call-backs or long wait times), difficulty using the platform independently, and limited phone access often sharing devices with CHWs, partners, or in-laws, or using inactive SIM cards. CHWs also noted that higher-income or working-class mothers were more likely to have health insurance or prefer private health services.

“Mostly, we would have encounters with mothers between 19 and 35 years who were willing to register more than those who are beyond 35 years. Those above 35 years old felt uncomfortable engaging the (virtual) doctors.” **FGD, CHW**

“For my registration, the younger pregnant women responded easily, as they don’t have pregnancy experience. They respond easily so that they can get help.” **FGD, CHW**

### Motivators and key enablers for client registration and client use

Overall, while the majority of the virtual CHWs delivered relatively well in enrolment, a strong backbone of performance management by supervisors was required to maintain momentum. CHW marketing of the virtual service primarily focused on the hotline as an emergency or clinical counselling service. Majority, 62% (24/39 CHWs) trained as virtual ambassadors onboarded a satisfactory level (10–28) of clients since project launch.

#### Enablers

Key drivers of successful performance include strong supportive supervision and confidence of certain CHWs to onboard and integrate registration as part of independent day-to-day activities; good levels of CHW technical proficiency (to guide households and handling and troubleshooting); and a clear understanding of roles and responsibilities. In addition, those that were successful with onboarding were overall well performing CHWs, with some of the factors that worked in favour included having a reliable means of transport which increased their reach and access, regular CHW engagements with clients to promote telehealth utilization, and CHWs focusing on key health areas such as pregnancy enabled them to perform higher than their counterparts.

**Figure 3:**
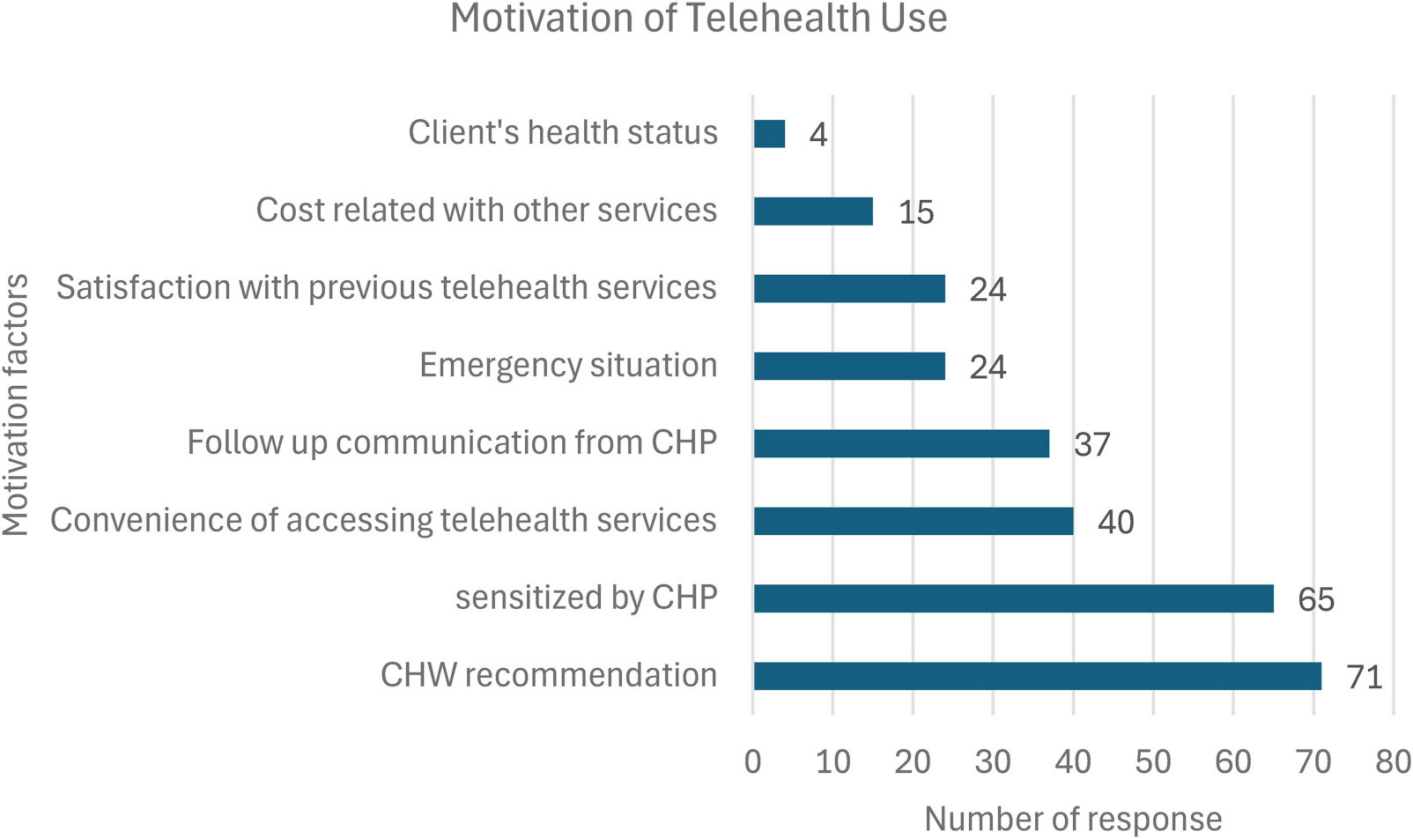
Motivation of telehealth Use.

During the pilot, we focused primarily on marketing channels through CHWs directly (Figure 3). However, insights from the FGDs suggest that clients who were initially resistant (mid—late adopters) to the service were influenced by peer recommendations and stories of the value of telehealth. According to our HealthX KII, the use of snowballing for pregnant mothers to refer other pregnant mothers for registration led to a surge in registration.

Peer Recommendations: “When we started the registration, most of them were unwilling. But the moment those who registered started engaging the telehealth doctors and got to see the benefits, they then came back and passed the message in the community. They became our good ambassadors, and this made our work easier. Those who are registered will go and tell the others.” FGD, CHW from Moding

#### Inhibitors

Barriers to the registration of clients to telehealth services included misconceptions about telehealth services, spousal refusal, lack of mobile phones, technical challenges, socio-cultural aspects, and privacy concerns (ie, disclosing a pregnancy early or HIV status). The low CHW performers reported that some clients held misconceptions about the purpose and efficacy of telehealth services, which led to hesitations in registering. In some households, women faced resistance from their spouses in utilizing telehealth services. In addition, limited access to mobile phones made it hard for them to access telehealth services.

**Figure 4:**
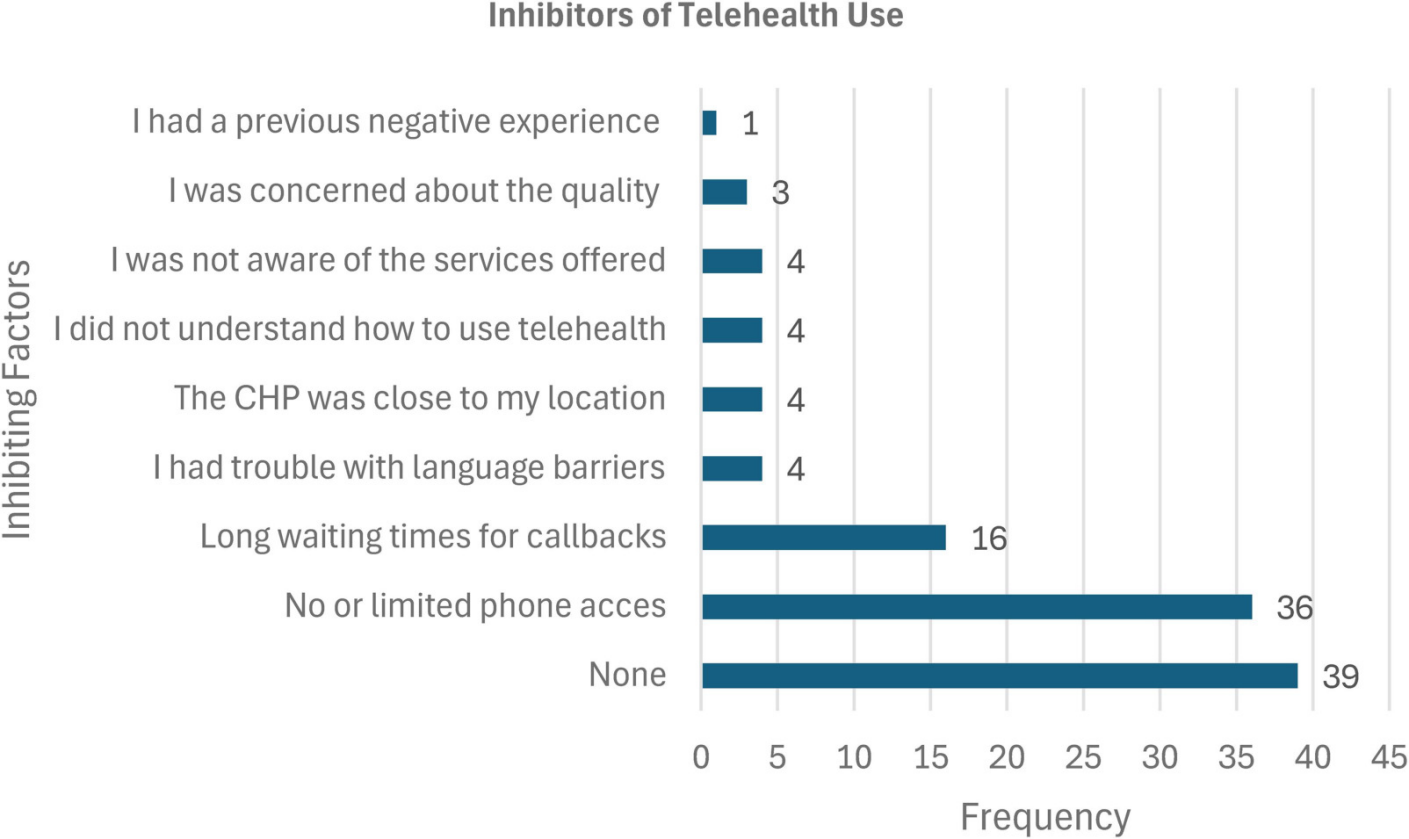
Inhibitors of telehealth Use.

Figure 4 shows the behavioral and contextual factors shaping telehealth adoption among mothers. Key barriers limited phone access, misinformation, and low digital literacy highlight structural inequities affecting inclusion. These results, consistent with focus group and survey data, emphasize that digital adoption is socially influenced, not purely technical.

### Uptake of services: functions & channels

Based on Health X consultation records, strong client preference for the toll-free clinical calls remains the predominant channel accessed and used by households, with very limited use of the other USSD or IVR enabled functions and features (hail your CHW, danger sign risk assessment, report a delivery). SMS reminders likely prompted positive behaviour change, with 64% (83/129) of women reporting the reminders improved their timely clinical ANC and PNC attendance. On exploring how the respondents used Telehealth. The majority of the respondents (*n* = 90, 83%) used the platform to speak to/consult a doctor when they or their baby were ill. This was followed by 68 respondents who reported registration of themselves or their baby on delivery, while very few (*n* = 6) used the platform to hail their CHW.

**Figure 5:**
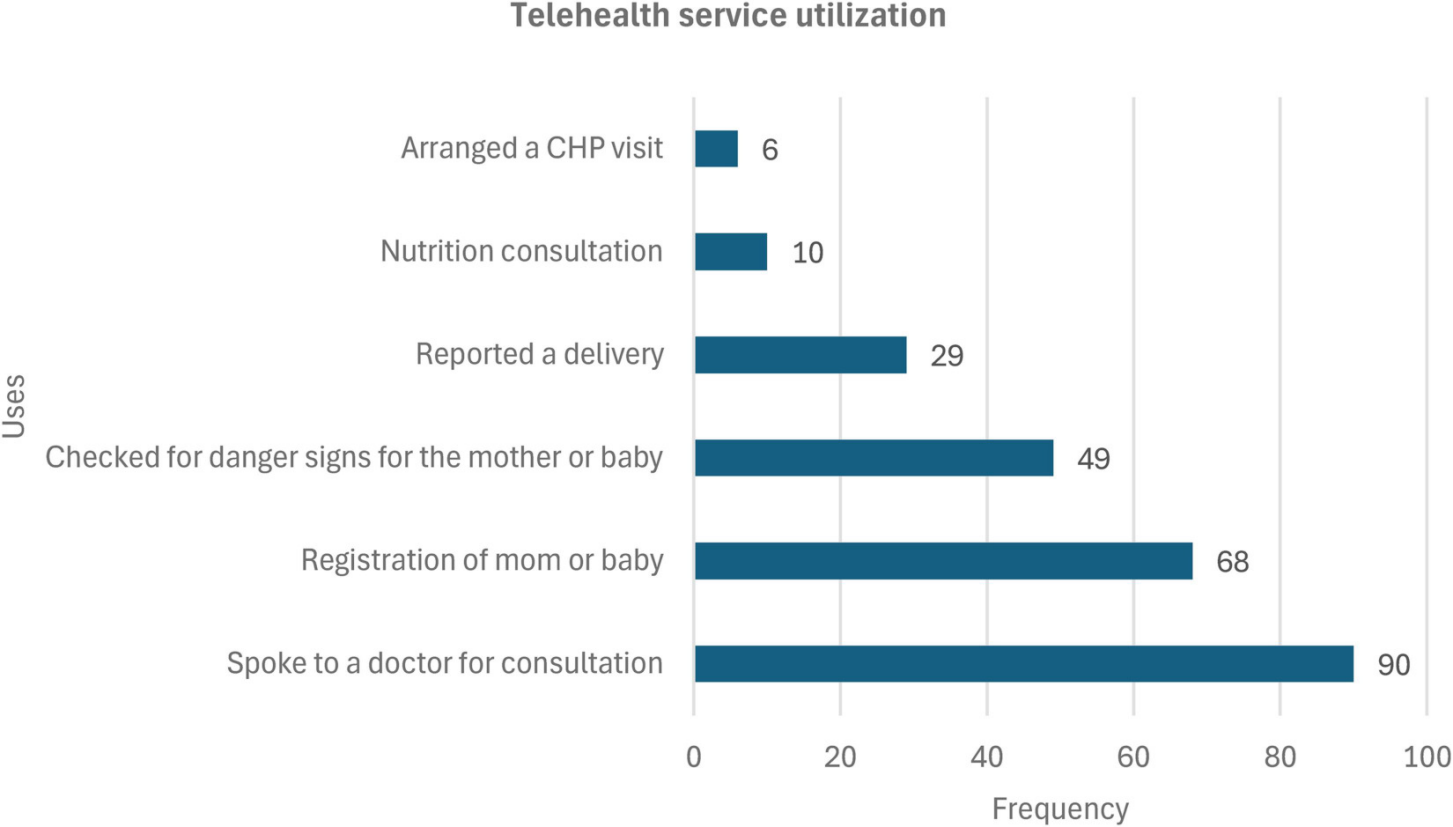
Telehealth services utilization.

Figure 5 illustrates utilization trends and service demand. The figure demonstrates that consultations for maternal and infant danger signs were the dominant use case (90%), confirming the model's relevance for emergency triage and maternal decision support. Secondary uses such as registration, follow-up, and nutrition consultations illustrate the gradual expansion of user engagement from reactive to proactive health behaviors. This progression aligns with the project's outcome framework, which aimed to move clients along a continuum from basic awareness to self-directed service use.

Both in IVR and USSD, the risk assessment tool (users can hear a list of danger signs associated with new mothers or infants in the PNC period) and the “Hail your CHW” had very limited uptake. In part early insights suggest this may have been due to a) low perceived value (clients already have the CHW phone number) b) a lack of active marketing by CHWs of these features c) that calling the virtual Doctor directly via the hotline was a more reassuring and direct experience for the user and d) that the virtual triage (e.g., danger signs) would escalate to a virtual Dr consultation).

### Telehealth impact

#### Management of high-risk cases and danger signs

Between June 2023 and September 2024, a total of 1,108 calls were made to the hotline, of which 61% (677 calls) were clinical encounters. The majority of these clinical consultations (78%, 529/677) were related to maternal health, while 22% (148/677) concerned infant care. Telehealth was valued by users as a confidential and accessible channel for seeking advice and support, especially for maternal health needs. Most clinical calls (64%, 435/677) were effectively managed by Health X without the need for referral, while 12% (79/677) required onward referral to local health facilities or specialists. Only six cases during this period required a prescription. Notably, 23% (248) of the clinical calls occurred outside of the standard 8 AM–5 PM CHW working hours, highlighting the extended access offered by telehealth services. High-risk cases accounted for 49% (327/677) of all clinical consultations, with the majority (83%, 272/327) involving pregnant women or mothers, compared to 17% (55/327) involving infants (Table 5).

**Table 5:** Clinical consultations risk analysis.

Risk analysis (clinical consultations) June 2023–September 2024						
Categorization	Baby #	%	Pregnant women & Mothers #	%	Total	%
Clinical consultations	148	22%	529	78%	677	
High risk	55	8%	272	40%	327	49%
Low risk	90	13%	249	37%	339	51%

Self-edited HealthX data.

#### Completion of referral

**Figure 6:**
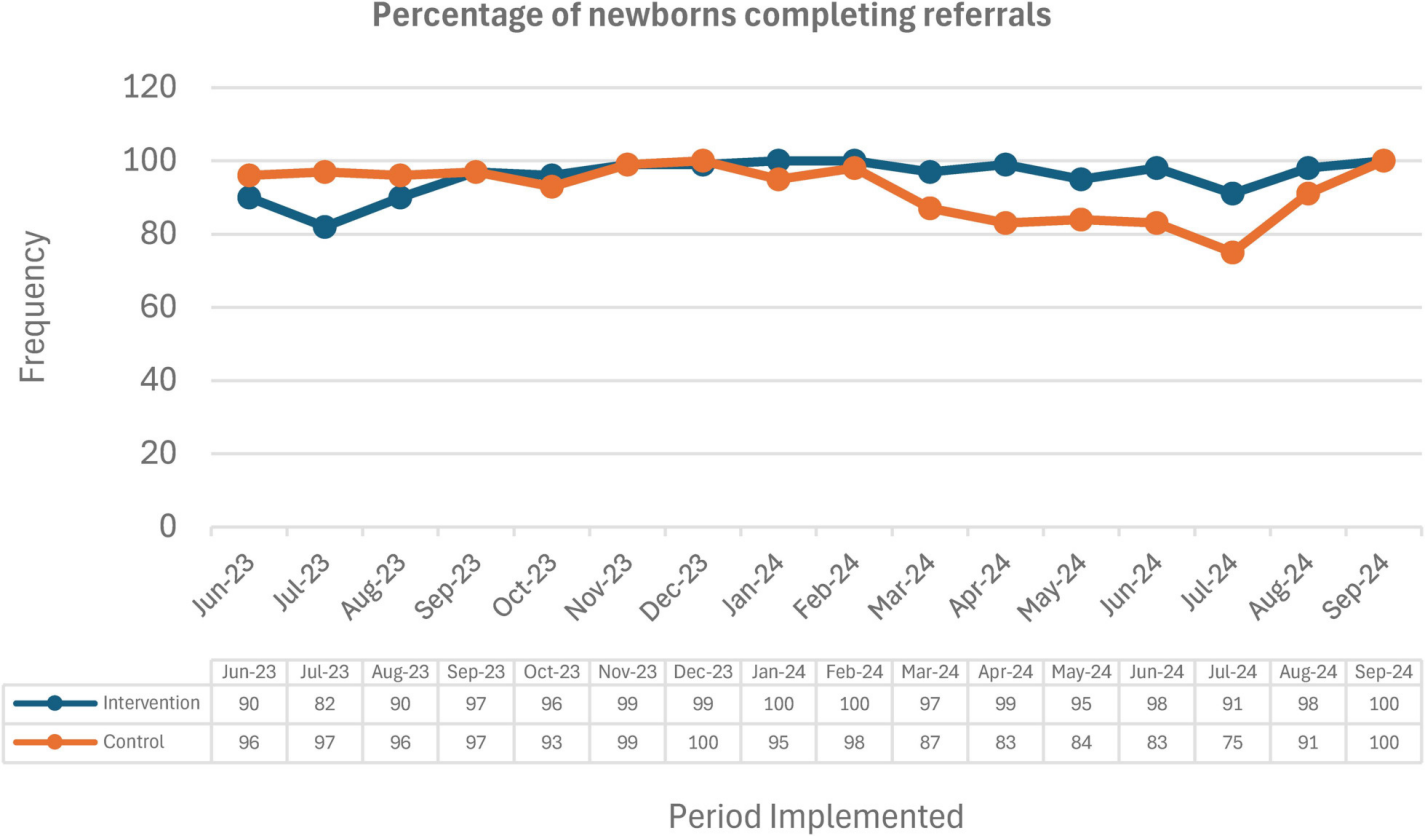
Percentage of newborns completing referrals.

Across the project duration (July 2023–September 2024), 92% (3,630/3,967) of referrals were completed in the intervention sites, compared to 97% (1,873/1,938) in the control site. While the control site had a higher percentage, the absolute number of completed referrals was substantially higher in the intervention sites (3,630 vs. 1,873). The report notes, ‘Based on the program data, control sites were referring to a higher number of infants with danger signs, the absolute loss to follow up in the control site was much higher at 329 infants compared to 62 infants in the intervention site’. This suggests the telehealth intervention effectively triaged cases, potentially reducing the burden on higher-level care facilities. Furthermore, the intervention sites achieved a consistently higher performance on proportion of completed referrals for infants with danger signs compared to the control site and maintained this positive trend even during periods of disruption, as shown in the Figure 6.

**Figure 7:**
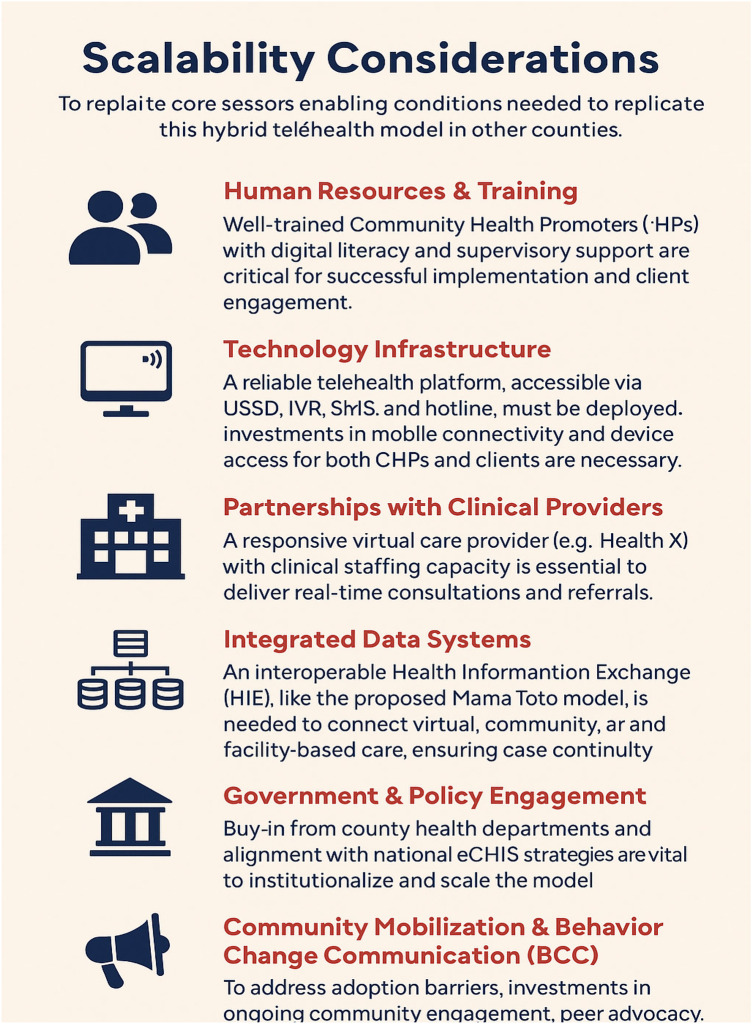
Scalability considerations.

FGDs and KIIS with CHP supervisors and CHPs revealed that the back-end SMS closed loop communication between CHP, Supervisor and Health X upon client referral supported timely follow up of both pregnant women, new mothers, and infants experiencing danger signs. Areas for critical improvement in the next version of the hybrid telehealth model should focus on leveraging the efficiencies of this early-stage testing of the closed loop referral follow up, including

Integrating the Health Facility within the referral communication systems
Integrating more aspects of end-to-end continuum of care so that clients can be more easily managed virtually (i.e., teleconsultant facilities at the health facility; introduction of local e-prescription sites and community-based distribution of prescription commodities, where appropriate).

Value of Health X and CHP communication on follow up: “The project benefited us because I was receiving reminder messages to go and visit my clients, and this simplified my work as a CHP. Such as a reminder to do a sickness follow-up.” FGD, CHP

### Number of PNC touchpoints

The average number of PNC touchpoints between CHW and client within 6 weeks of delivery was, on average, statistically (*p*-value: 0.003) slightly higher in the intervention site at approximately 5 visits (4.99) compared to approximately 4 visits (3.96) in the control community units. However, these averages mask a greater monthly fluctuation of visits in the intervention sites during pilot implementation. Using the 20% treamed means and Winsorized standard deviations, the generalized Cohen's d comparing the intervention and control was 3.67 (95% CI: 1.64;8.02), indicating a very statistically meaningful effect. This implies Even after accounting for non-normality and outliers, the Intervention produced a substantial difference in with six weeks compared to the Control.

“Those women who were registered on Telehealth after delivery know the best practices on how to take care of a newborn. Those who were not registered, if the CHW has not also coached them correctly, don't know the best practices for newborn care. They have enough education on newborn birth care practices. Those on telehealth, they talk, they speak to the doctors frequently, unlike those who are not registered, so they tend to get more knowledge than those who are not registered.” **FGD, CHW**

Survey with mothers revealed that the experiment group, on average, has a significantly higher number of PNC visits (4.99) compared to the control group (3.96) but with more variability. Hence, while the intervention in the experimental group may lead to more visits on average, the results are more variable and less consistent. Across June 2023–September 2024, trends for the unique women/infants visited at least once by their CHW within 48 h were on average 85% compared to the control of 87%. Women/infants receiving all three PNC touchpoints’ on time within 6 weeks, was not positively impacted by the virtual platform when we observe the trends between intervention and control site (334/625 (53%) vs. 965/1589 (61%), however due to the difference in the total number of women being served, the intervention site experienced significantly fewer cases of women/infants lost to follow-up during that period (291 compared to 625).

### Determinants of on time PNC

Three variables in Table 6 emerged as statistically significant predictors of on time PNC: previous PNC status (LAG PNC), previous follow-up activity (LAG follow up counts), and maternal age.

**Table 6:** Determinants of on time PNC visits.

Population-averaged logistic regression model using generalized estimating equations (GEE)			
Variables		Estimates (95% CI)	*P* value
Community units	Control units	1	
	Intervention units	0.81 (0.22; 2.96)	0.975
Follow up counts		0.41 (0.07; 2.28	0.308
ANC counts		1.86 (0.86; 4.02	0.118
Patient age		1.16 (1.04;1.29)	0.007
LAG follow up counts		9.71 (1.08; 86.91)	0.042
LAG ANC counts		0.53 (0.26; 1.06)	0.072
LAG patient age		0.97 (0.91; 1.29)	0.047
LAG PNC		55.21 (7.00; 445)	<0.01

First, prior on time PNC status was the strongest predictor of current PNC uptake. CHWs who had done timely PNC visit in the previous period had markedly higher odds of doing another timely PNC visit again (OR = 55.21; 95% CI: 7.00–435.59; *p* < 0.001). This substantial temporal persistence suggests that timely PNC may reflect underlying behaviors and interactions patterns that CHW continue over time. Once a CHW woman engages successfully with the woman during the postnatal care, this behavior appears highly likely to repeat.

Second, higher follow-up activity in the previous time period was associated with increased odds of on time PNC in the current period (OR = 9.71; 95% CI: 1.08–86.91; *p* = 0.042). Although the confidence interval is wide, indicating variability, the direction and significance suggest that consistent or intensified CHW engagement may help facilitate timely PNC in subsequent periods.

Third, maternal age was positively associated with on time PNC, with each additional year of age increasing the odds of timely PNC by approximately 16% (OR = 1.16; 95% CI: 1.04–1.29; *p* = 0.007). This indicates that younger mothers remain a high-risk group for missed or delayed PNC services.

### Perceived value and impact for CHWs

According to FGDs and KIIs with CHWs and Supervisors, the introduction of the hybrid virtual model resulted in several system efficiencies and benefits:

Improved follow-up by making it easier for CHWs to monitor pregnant and post-partum mothers through notifications
Boosted CHWs’ confidence and their standing within the community through the association with Health X doctors,
Reducing the need for extensive education during sessions, with registered clients more likely to be receptive and knowledgeable with positive health-seeking behaviours
Telehealth simplified referrals and disease identification, making it easier for CHWs to refer clients to health facilities, and streamlined follow-up tasks by alerting CHWs to client needs.
Health X doctors assisted CHWs in encouraging clients to visit health facilities when referred.
Provided a way to more immediately escalate a health concern from the household

For the highly adopting clients and the highly performing CHWs, the KIIs and FDGs revealed a level of promising synergy between client, CHW, and virtual telehealth solutions, as intended in the original design:

“The virtual support brought the clients closer to me during the ANC period. Each time the client engages the telehealth doctor, they would call me, and any time I received SMS about a certain client, I would visit them… I would say 90% drew closer due to the virtual support,” FGD, CHW High Adopter.

“…For those (clients) who do not read their messages, I communicate with them as soon as possible and pass the message to the relevant personnel in Telehealth. This has helped to facilitate faster communication,” FGD, CHW.

## Discussion

This study evaluated the integration of telemedicine into Kenya's CHW framework to improve MNCH outcomes. The findings highlight the critical role of CHWs in facilitating telehealth adoption, the importance of early maternal engagement, and the influence of community-driven adoption patterns on service utilization. While the intervention demonstrated significant improvements in healthcare access, sociocultural barriers, program awareness, and CHW capacity remain key challenges to long-term sustainability and scale.

### Enhanced access and utilization of maternal health services

The telehealth intervention exceeded its registration targets, enrolling 388 households and 551 clients, demonstrating strong community engagement and improved access to maternal and newborn health services. The significant proportion of women enrolling in their second trimester indicates that the platform effectively encouraged earlier engagement in antenatal care. Moreover, the observed increase in postnatal care (PNC) touchpoints reflects a positive shift toward consistent maternal and neonatal health utilization, aligning with evidence from a retrospective study that reported similar increases in PNC attendance and postpartum depression screening following telemedicine adoption (8).

Qualitative findings from the FGDs and KIIs highlighted significant socio-cultural barriers that influenced women's ability to fully participate in virtual consultations. Gender dynamics were particularly pronounced, with reports of spousal refusal, male control over household phones, and restrictions on women's independent decision-making, patterns consistent with broader evidence showing that women often have limited agency over mobile phone access and use in low-resource settings. Low general and digital literacy further constrained women's confidence and capacity to navigate teleconsultation platforms, echoing previous studies demonstrating how literacy gaps disproportionately affect women's adoption of digital health tools (9). Together, these findings underscore the importance of designing telemedicine interventions that explicitly address gender norms, household power dynamics, and literacy barriers to ensure equitable access and meaningful participation.

Comparable findings have been documented in other African contexts, further validating the role of telehealth in improving maternal and child health outcomes. In Uganda, the *Safe Delivery mHealth* project demonstrated significant increases in skilled birth attendance and timely referral completion through mobile-based decision-support tools for community health workers (10). Similarly, in Nigeria, the *HelloMama* and *Smart Start* mHealth initiatives improved antenatal visit compliance and immunization uptake by providing structured voice and SMS messaging tailored to maternal needs (11). The use of mobile health tools by community health workers in sub-Saharan Africa was linked to higher uptake of antenatal care, more facility-based deliveries, and improved postnatal care attendance. To maximize the impact of these programs, it is important to recognize and address both the social and technical challenges that affect mHealth adoption (12).

Evidence from other continents also supports these trends. In Bangladesh, the *Aponjon* mobile health service reported increased antenatal care attendance and early postpartum check-ups, largely driven by personalized mobile reminders and remote health counselling (13). In Peru, telemedicine-supported maternal care in rural Andes communities enhanced follow-up adherence and early complication detection, demonstrating the effectiveness of hybrid care models in geographically challenging environments (14). Models such as “Mobile for Mothers” and the ReMIND mHealth intervention in India demonstrate that digital solutions can expand access to antenatal, intrapartum, and postnatal care, especially for low-literacy users. These studies emphasize that digital innovations must be carefully conceptualized, co-designed with communities, and rigorously evaluated to ensure equity and reach among vulnerable populations (15).

Collectively, these studies complement the Kenyan telehealth model's findings by confirming that hybrid, community-linked digital interventions can bridge geographical and socioeconomic barriers, improve service continuity, and promote proactive health-seeking behaviors. The convergence of evidence across Africa, Asia, and Latin America underscores telemedicine's global potential as a transformative enabler of equitable maternal and newborn care.

### Improved referral completion and follow-up

The intervention achieved a high referral completion rate of 92%, with significantly more referrals completed in intervention sites compared to control sites (3,630 vs. 1,873). Although control sites exhibited a slightly higher percentage of completed referrals (97% vs. 92%), they also recorded a larger number of missed referrals (329 vs. 62), indicating that telehealth facilitated more efficient triage, case prioritization, and timely follow-up. This efficiency likely contributed to reduced facility congestion and improved continuity of care for maternal and neonatal clients.

These findings align with previous evidence showing that telehealth interventions can strengthen referral linkages and continuity of care by enabling faster communication and remote monitoring during prenatal and postnatal periods (16). Similar outcomes have been reported in Ethiopia, where an mHealth referral tracking system increased referral completion among community health workers, real-time data exchange and timely pregnancy registration contributed to increased utilization of maternal health services across all stages of care (17), and in Ghana, where mobile decision-support tools improved timely referrals for obstetric emergencies (18).

SMS reminders and digital alerts also played a vital role in promoting appointment adherence and proactive client engagement. Evidence from Tanzania demonstrated that automated SMS reminders increased antenatal visit attendance by 30% (19), consistent with findings by Knop et al. (20), where targeted SMS interventions improved timely child immunization rates. Collectively, these results underscore that mHealth-enabled communication channels such as SMS, IVR, and teleconsultation platforms can effectively bridge service delivery gaps, improve follow-up adherence, and enhance patient-centered care in resource-limited settings. This reflects how behavioral nudges and low-cost communication tools can reinforce patient accountability and care continuity within telehealth ecosystems.

### Effectiveness of the hybrid telehealth model

The study highlights the effectiveness of a hybrid telehealth model that integrates virtual consultations with community-based support from CHWs, fostering trust, community ownership, and continuity of care. However, challenges in integrating telehealth into routine CHW workflows underscore the need for sustained digital capacity-building, human-centred design, and supportive supervision (11). This aligns with broader evidence from Africa indicating that telemedicine can improve maternal outcomes, while persistent barriers such as infrastructure limitations and digital literacy gaps continue to constrain scale-up (8).

Complementary evidence from maternity care during the COVID-19 pandemic demonstrates that blended telehealth approaches, combining virtual group-based education with individual antenatal assessments, can be successfully embedded within routine clinic workflows. Buultjens et al. show that a hybrid, interdisciplinary telehealth model supported maternal psychological wellbeing when delivered by a small but committed clinical team, emphasizing the importance of continuity, relational care, and structured group engagement rather than telehealth delivery alone (21). Published in Women and Birth, this work reinforces the view that telehealth functions most effectively as part of a broader digital care ecosystem, rather than as a stand-alone operational substitute for in-person services. Together, these findings extend earlier telemedicine literature by positioning hybrid telehealth models as catalysts for health system redesign, enabling innovation diffusion, workforce role evolution, and patient empowerment. Comparable experiences from Indonesia, South Africa, and India further demonstrate that telemedicine success depends not only on technical infrastructure, but also on leadership support, digital adoption capacity, and user engagement frameworks (6, 20). For instance, the Indonesian hospital digital adoption model highlights how innovation readiness and data interoperability determine service performance in digitally transforming hospitals (20). Similarly, the study on Exploring consumer sentiments in telemedicine identifies that trust, perceived utility, and human connection drive sustained engagement (6). These insights expand the interpretation of Kenya's findings beyond access metrics, positioning telehealth as part of a larger digital innovation continuum that links community health with institutional transformation. Parallel experiences across Africa reinforce this trajectory. A randomized trial in Kenya and Uganda found that CHW-facilitated telehealth for hypertension achieved 86% control rates at 48 weeks, outperforming clinic-based care (22). In Malawi, CHWs adopting mHealth tools reported improved confidence and efficiency, though persistent barriers included connectivity and training gaps (22).

Systematic reviews also affirm telemedicine's benefits for chronic disease management, showing significant improvements in HbA1c and systolic blood pressure outcomes (25). Such global comparisons position telehealth as not only a service delivery tool but as a foundation for patient-centered digital transformation. These findings align with work showing that telehealth enhances chronic-disease management particularly diabetes by improving health outcomes, patient satisfaction and accessibility (3). Notably, in the hybrid model studied, the high reliance on hotline consultations as a preferred communication channel underscores the feasibility of integrating telehealth into community-health-service delivery. This alignment suggests that pairing digital consultations with trusted CHWs and local support systems offers a promising pathway for scale. Nonetheless, successful deployment at scale will require addressing device access, connectivity, training, supervision, data systems and sustainable funding models.

### Addressing healthcare access barriers

Comparable telehealth experiences from other low- and middle-income countries further demonstrate that blended digital–community health models can be successfully scaled. Telemedicine has become a central component of contemporary healthcare systems (3), especially as countries sought alternative care pathways during the COVID-19 pandemic. In India, the expansion of these services has been slow, largely because healthcare consumers have not adopted them at the expected rate (7). In Indonesia, platforms such as Halodoc and Alodokter supported millions of remote consultations, helping ease pressure on already strained health facilities (24). Likewise, Bangladesh and India (7) rapidly broadened public access to telemedicine services during the pandemic, demonstrating how digital health tools can maintain service continuity when in-person care is disrupted. Indonesia's *Digital Midwife* and the Philippines’ *National Telehealth Service Program (NTSP)* demonstrate how digitally supported CHWs extend clinical reach to underserved regions (25, 26). These experiences mirror the Kenyan pilot's outcomes, illustrating a shared path toward digitally enabled, equity-focused primary healthcare. Based on the findings of the studies, the initiative effectively reduced access barriers by allowing remote consultations, minimizing travel costs and delays. However, sociocultural dynamics, gender norms, and connectivity inequalities limited enrolment among certain groups. The study also emphasizes that bridging these inequities requires inclusive design, digital literacy training, and integration with national infrastructure to ensure equitable service reach.

In this study, the telehealth initiative effectively tackled healthcare access challenges in remote and underserved communities. The ability to consult healthcare providers via mobile phones reduced the need for physical travel, addressing both cost and distance barriers. However, sociocultural factors such as spousal approval and limited program awareness impacted enrolment rates, particularly among certain demographics. The study also identified gaps in phone access and network connectivity, which need to be addressed to ensure equitable access to telehealth services. This study supports the growing evidence that adopting telehealth technologies can enhance the antenatal care experience for women and improve access, while potentially reducing healthcare costs without negatively affecting maternal or neonatal health outcomes. However, as highlighted in recent literature (e.g., *Telehealth in antenatal care: recent insights and advances*), further research is needed to assess the impact of telehealth on rare but critical outcomes such as maternal and neonatal mortality (27). Additionally, the study echoes concern from (28) Berkley et al., which highlight that many high-risk children go undetected while low-risk children often receive unnecessarily intensive care. This imbalance underscores the importance of integrating telehealth solutions to ensure timely identification and escalation of high-risk cases, ultimately optimizing healthcare resource allocation and improving survival outcomes.

### Policy and practical implications

This study aimed to produce evidence that can inform future telehealth policy decisions, particularly those related to the broader socioeconomic effects of virtual care. Although rapid technological progress has strengthened telehealth delivery and motivated the development of federal reimbursement proposals, legislative action has slowed due to ongoing concerns among policymakers. Because policy debates often move quickly, decision-makers require timely, synthesized reviews of the most reliable evidence to determine whether telehealth legislation should be advanced, amended, or withheld (29). This research responds to that need by supplying relevant insights to support informed deliberation.

**Optimize a digitized closed loop referral system (CLR):** A comprehensive, integrated digital platform that links CHWs, supervisors, telehealth clinicians, and health facilities would streamline referral initiation, triage, tracking, and completion. Such a system would reinforce shared decision-making, reduce uncertainty in referral pathways, and improve feedback mechanisms to both clients and providers. By connecting all stakeholders in real time, a digitized CLR would support seamless coordination, enhance continuity of care, and advance a more responsive, data-driven telehealth ecosystem. Families often emphasize that high-quality referral decisions rely on collaborative communication and clear, specific information such as a shared understanding of why the referral is needed and which provider or subspecialist the client should see. Challenges arise when frontline providers experience uncertainty about whether referral is warranted or lack clarity on the most appropriate specialist for the case, which can limit meaningful shared decision-making and hinder timely escalation of care (30). Within our telehealth pilot, the hybrid model helped address several of these barriers by enabling CHWs and clients to access real-time clinical support through the hotline, improving clinical confidence and the precision of referral decisions. However, to fully capitalize on these gains and ensure consistency across all service delivery points, it is essential to strengthen the system infrastructure for managing referrals.
**Ensure interoperable client data:** Ensuring the interoperability of client records is essential for strengthening telehealth integration within primary healthcare systems. Linking community-level data streams to Kenya's national electronic Community Health Information System (eCHIS) would enable a more comprehensive, data-driven approach to identifying and supporting high-risk women and infants. This aligns with the WHO Global Strategy on Digital Health (2020–2025) (31), which emphasizes the need for inclusive, interconnected digital health ecosystems. Yet many low- and middle-income countries continue to face structural constraints including unreliable digital infrastructure, fragmented governance arrangements, and weak regulatory oversight that limit progress from pilot-level innovations to fully operational telehealth solutions. A major barrier is the lack of system interoperability; as highlighted by the Healthcare Information and Management Systems Society, limited ability for platforms to communicate seamlessly impedes broad telehealth adoption (32). Although interoperable electronic health records have the potential to enhance coordinated, value-based care, persistent gaps in usability and clinician satisfaction suggest the need for both organizational and provider-level interventions to reduce inequities in digital access (33). Looking ahead, strengthening platform security will also be critical. Integrating AI-driven threat detection and adopting quantum-resistant encryption approaches can offer more robust protection as telehealth systems expand and the cybersecurity landscape grows increasingly complex (34).
**Use data for risk identification & stratification:** Pilot and refine risk stratification models using client data, including testing predictive algorithms and applying both low-tech and AI-enabled diagnostics to identify high-risk women and children under two years of age. Client data and predictive models, including AI diagnostics, help identify high-risk women and children under two, improving targeted care. AI-enabled telehealth enhances access and quality, especially in underserved areas, by automating assessments, supporting decisions, and enabling remote monitoring. Sustainable models like subscriptions and public–private partnerships can scale these solutions while aligning incentives with outcomes. Ethical, regulatory, and technical challenges data privacy, algorithmic fairness, and transparency must be managed to maintain trust. Risk assessment tools optimize resource allocation for large populations (35), and precision prevention leverages digital health and AI to provide personalized interventions, as seen in cardiovascular disease prevention, while addressing equity and system-level concerns (9).
**Deliver personalized & precision care at the household level:** Enrolled clients into customized care pathways based on their health profiles, supported by targeted notifications (e.g., IVR, SMS), virtual consultations, and CHW-led services tailored to their specific needs. Building on evidence from global telehealth studies, Similarly, delivering pediatric subspecialty care in local clinics or community hospitals has been shown to improve adherence to evidence-based guidelines, enhance provider, patient, and family satisfaction, and reduce costs, while addressing geographic and care-quality disparities in underserved populations (36). However, as highlighted in broader telehealth research, the effectiveness of these interventions is constrained by the digital divide, which limits access and digital literacy among vulnerable groups. Expanding technology access and digital skills, combined with equity-focused policies addressing social determinants of health, is essential to ensure inclusive, effective, and scalable maternal and child health telehealth solutions (37).
**Scalability considerations for hybrid telehealth**: Scalable hybrid telehealth requires trained frontline workers, reliable digital platforms, strong clinical partnerships, interoperable data systems, committed government involvement, and active community engagement (Figure 7). Improving adoption also depends on human-centered design, aligning technology with user needs, and addressing infrastructure, privacy, and interoperability challenges (38). Evidence from recent reviews shows that scalability assessments often overlook important contextual and technological domains, and varied evaluation methods limit comparability across studies. Future research should identify the essential domains and standardized methods needed to evaluate the scalability of remote telemonitoring and telehealth services to better guide large-scale implementation efforts (39).

This pilot demonstrated the feasibility and impact of integrating telemedicine into community health structures to improve maternal and newborn care in underserved settings. The hybrid model combining virtual doctor consultations, CHWs, and SMS-based engagement enhanced access, improved referral completion, and supported positive health-seeking behaviours. Notably, insights from this pilot have already informed early discussions with county health officials regarding the integration of telemedicine into routine MNCH programming. As part of this engagement, stakeholders are exploring a closed-loop solution which connects virtual care (via the telemedicine provider) with physical care (CHWs and health facilities) through a Health Information Exchange (HIE), ensuring seamless data sharing, case continuity, and patient-centered care across the continuum.

## Lessons learnt

**Leverage CHWs as central catalysts**: CHWs with strong technical skills, community trust, and supportive supervision played a pivotal role in driving adoption and engagement with telehealth. Future rollouts should prioritize training, motivation, and digital literacy support for CHWs.
**Start with strong virtual customer service**: Early user experiences with the hotline significantly influenced platform perception. Timely callbacks, respectful tone, and continuity of care should be non-negotiable standards.
**Target first-time and high-risk mothers**: These groups showed the highest uptake, indicating a natural demand for personalized, remote support. Tailoring messaging and outreach toward them can boost adoption.
**Enable peer-to-peer influence**: Snowball referrals and positive word-of-mouth among mothers were powerful adoption drivers. Incorporate structured community advocacy and testimonial-based messaging into future strategies.
**Simplify tech tools and messaging**: Features like “Hail your CHW” or IVR self-screenings saw low uptake due to complexity or poor awareness. Ensure user-centered design, ongoing promotion, and clarity of value in future rollouts.
**Address device and access barriers**: Limited phone access and shared devices were major barriers, especially for adolescent or low-income mothers. Future designs should explore shared device protocols, offline functionality, and equity strategies.
**Avoid overreliance on one channel**: The hotline dominated usage, while other channels (e.g., USSD, IVR) underperformed. Balance promotion efforts across channels to maximize the platform's full potential.
**Plan for sociocultural resistance**: Factors like spousal refusal, privacy concerns, and stigma delay uptake. Proactive community engagement, male partner sensitization, and anonymous or discreet features should be embedded in design.

## Limitations and future research

While the study provides valuable insights into the potential of telemedicine in community health, several limitations should be noted. The purposive selection of the intervention (4 CHUs) and comparison (4 CHUs) sites introduces the possibility of selection bias, which limits the strength of causal inference. However, this approach was appropriate given that the primary objective of the study was to assess feasibility, adoption, and contextual effectiveness, an essential precursor to designing a large-scale randomized trial. Further, the short pilot duration, is insufficient to assess long-term adoption, financial sustainability, or the potential for permanent institutionalization of the hybrid telemedicine model within the county health system.

Future research should incorporate more rigorous evaluation methods, including randomized control groups and robust outcome measures, to better assess the long-term impact of telemedicine interventions on MNCH outcomes. Further investigation into cost-effectiveness, sustainability, and the feasibility of integrating telehealth into national health systems will also be critical in informing scale-up in similar contexts. And finally, future evaluations should include longer follow-up to address the aspects of long-term outcome assessments.

## Conclusion

The ‘*Better Data for Better Decisions’* telemedicine initiative in Kenya demonstrated promising results in enhancing maternal and newborn child health services by integrating technology with the existing community health worker framework. The project exceeded registration targets, and the use of hotline consultations and SMS reminders facilitated increased postnatal care touchpoints and improved appointment adherence. While the study highlights the potential of this hybrid model to strengthen follow-up and referrals, further investigation is needed to quantify the impact on overall healthcare delivery efficiency and long-term policy influence. Insights from this pilot have already informed early discussions with county health officials regarding telemedicine integration into routine MNCH programming.

## Data Availability

The original contributions presented in the study are included in the article/Supplementary Material, further inquiries can be directed to the corresponding author.

## Footnotes

^1^Interactive voice response is a technology that allows users to interact with a computer-operated telephone system through voice commands of keypad inputs. It enables callers to receive or provide information and make requests without needing to speak to a live agent.
